# Modulation of Brain Activity with Noninvasive Transcranial Direct Current Stimulation (tDCS): Clinical Applications and Safety Concerns

**DOI:** 10.3389/fpsyg.2017.00685

**Published:** 2017-05-10

**Authors:** Haichao Zhao, Lei Qiao, Dongqiong Fan, Shuyue Zhang, Ofir Turel, Yonghui Li, Jun Li, Gui Xue, Antao Chen, Qinghua He

**Affiliations:** ^1^Faculty of Psychology, Southwest UniversityChongqing, China; ^2^School of Education, Guangxi UniversityNanning, China; ^3^Department of Information systems and Decision Sciences, College of Business and Economics, California State University, FullertonFullerton, CA, USA; ^4^Key Laboratory of Mental Health, Institute of Psychology, Chinese Academy of SciencesBeijing, China; ^5^National Key Laboratory of Cognitive Neuroscience and Learning, IDG/McGovern Institute for Brain Research, Beijing Normal UniversityBeijing, China; ^6^Southwest University Branch, Collaborative Innovation Center of Assessment toward Basic Education Quality at Beijing Normal UniversityChongqing, China

**Keywords:** transcranial direct current stimulation (tDCS), drug addiction, Alzheimer's disease, major depression disorder, safety, decision neuroscience, cognitive neuroscience

## Abstract

Transcranial direct current stimulation (tDCS) is a widely-used tool to induce neuroplasticity and modulate cortical function by applying weak direct current over the scalp. In this review, we first introduce the underlying mechanism of action, the brief history from discovery to clinical scientific research, electrode positioning and montages, and parameter setup of tDCS. Then, we review tDCS application in clinical samples including people with drug addiction, major depression disorder, Alzheimer's disease, as well as in children. This review covers the typical characteristics and the underlying neural mechanisms of tDCS treatment in such studies. This is followed by a discussion of safety, especially when the current intensity is increased or the stimulation duration is prolonged. Given such concerns, we provide detailed suggestions regarding safety procedures for tDCS operation. Lastly, future research directions are discussed. They include foci on the development of multi-tech combination with tDCS such as with TMS and fMRI; long-term behavioral and morphological changes; possible applications in other research domains, and more animal research to deepen the understanding of the biological and physiological mechanisms of tDCS stimulation.

## Introduction

Brain activity is based on electronic firing of neurons. Thus, the possibility of being able to modulate, facilitate or disrupt this electric activity is appealing; it can help with creating temporary or somewhat permanent desirable brain changes. Various ways to achieve these goals, all of which focus on transcranial stimulation have been developed over the years. These include transcranial magnetic stimulation (TMS), transcranial direct current stimulation (tDCS), transcranial alternating current stimulation (tACS), transcranial random noise stimulation (tRNS), transcranial pulsed current stimulation (tPCS), and transcranial ultrasound stimulation (TUS) (Chen et al., 1997; Paulus, 2011; Tufail et al., 2011; Jaberzadeh et al., 2014). Among these methods, TMS and tDCS have been widely adopted and used in both healthy and clinical samples. This study focuses on tDCS and reviews its theoretical underpinnings, its uses and applications, outcomes and risks. Reflecting on these issues, this study provides recommendations for alleviating risks in future tDCS studies.

tDCS is a tool to induce neuroplasticity and modulate cortical functioning by applying weak direct current over the scalp of participants (Stagg and Nitsche, 2011). It has been widely used over the past decade, and has made significant contributions in the field of neuroscience and psychology (Fregni and Pascual-Leone, 2007). It is a noninvasive neuro-modulatory technique, which can reduce bidirectional polarity-dependent changes in underlying cortical areas. It can operate as both an exciter or an inhibitor of brain activity in regions of interest. Specifically, anodal stimulation can increase the excitability of such regions, whereas cathodal stimulation diminished it (Nitsche and Paulus, 2000; Antal et al., 2003). In addition, the electric current applied by tDCS can modulate the level of membrane potential as well as the firing rates of targeted cortical neurons (Nitsche and Paulus, 2000). The effects of tDCS stimulation can be long-lasting; the durability of such effects is a function of the duration and magnitude of the applied current. For example, it has been shown that the after-effect of a 13-min continuous stimulation can last up to 90 min (Nitsche and Paulus, 2001).

Given the persistent and possibly clinically useful effects of this noninvasive brain stimulation technique, tDCS has been utilized by clinicians and neuroscientists to treat mental and neurological disorders. Early attempts are dated to the nineteenth century. For instance, in 1804, the Italian physicist Giovanni Aldini successfully treated melancholic patients using electronic stimulation (Aldini, 1804). Later, Erb (1886) combined tDCS and muscle faradization to rehab motor function in chronic stroke patients. However, in the 1930s, the electroconvulsive therapy by Cerletti and Bini (1938) which aimed at helping patients with severe schizophrenia has overshadowed tDCS; this has led to the relative loss of interest in tDCS for 30 years (Utz et al., 2010). The interest in tDCS was regained in the 1960s, but was later neglected due to inconsistent results in human trials (Wagner et al., 2007). These inconsistencies can be attributed to difficulties in quantifying the stimulating current densities, electrode configurations, duration of stimulation, and/or frequency of stimulation (Barker, 1994; Nitsche and Paulus, 2000). Over the last decade, vast developments in neuroscience have brought new life to tDCS; it has been suggested that tDCS can be a potential treatment for drug addictions (Conti and Nakamura-Palacios, 2014; Wang et al., 2016), strokes (Hummel et al., 2006), epilepsy (Fregni et al., 2006d), Parkinson's disease (Fregni et al., 2006b), chronic pain (Fregni et al., 2007), Alzheimer's disease (Ferrucci et al., 2008a), and depression (Nitsche et al., 2009).

Despite the recent wide use of tDCS in a number of studies, the underlying mechanisms of the cortical excitatory/inhibitory effect of tDCS have not yet been fully understood (Arul-Anandam and Loo, 2009; Bikson et al., 2009). Furthermore, concerns regarding the safety of clinical treatment with tDCS have been raised. It is therefore important to synthesize knowledge regarding how tDCS works, when it works and risks associated with its use, in order to facilitate a more focused and safer use of tDCS in future research. To this end, in this review, we first introduce the basics of tDCS techniques, their protocols and typical montages. Then, the potential underlying mechanisms of tDCS, as well as the advantages of tDCS over other invasive and non-invasive brain stimulation techniques are discussed. Next, we describe key clinical applications among various populations, such as drug addicts, people with major depression disorder, and Alzheimer's disease. Following the description of tDCS applications, we discuss possible adverse effects of tDCS and safety procedures; these aspects are emphasized in relation to the use of tDCS in children. Lastly, potential directions for future research are proposed. These mainly focus on combining tDCS with other neuroscience techniques and extending the use of tDCS to other research domains.

## tDCS basics

### tDCS protocols

Most tDCS studies we reviewed have adopted similar stimulation protocols, which we describe below. The protocol typically includes applying a pair of sponges (about 25–35 cm^2^) soaked with approximately 6 ml saline solution (in rare cases, water) to stimulate the target site around 20 min with direct currents of 1–2 mA. The electrodes used in tDCS are typically a pair of metal or conductive rubber wrapped in a perforated sponge pocket. The sponges are soaked with saline solution to minimize skin resistance. An alternative choice that is occasionally employed is the use of rubber electrodes with conductive gel. The size of such electrodes is typically 25–35 cm^2^. Electrode positioning is usually determined according to the international 10–20 electrode placement system. Constant current of 1–2 mA is delivered to the subject's scalp through anodal and cathodal electrodes with a ramp up and ramp down period of 30 s at the start and end of the session. Researchers have demonstrated that the after-effect of 10–30 min tDCS stimulation could last for about 1.5 h (Nitsche and Paulus, 2000; Nitsche et al., 2003a).

### Potential montages

Montages refer to the potential configurations of the electrodes; different arrangements and placements produce different results as they send currents through and stimulate different brain regions. Bi-cephalic tDCS is a common montage. It involves a cathodal electrode which decreases the function of the targeted brain areas whereas the anodal electrode increases this function (Nitsche and Paulus, 2000; Nitsche et al., 2003b). However, there can be variability in the placement and configuration of electrodes and this can result in different, and sometimes opposite results. For example, Moliadze et al. (2010) provide some of the strongest clinical evidence to-date that the relative position of stimulation can affect neuromodulation under each electrode. Moreover, increasing electrode distance may decrease the magnitude of neuro-modulation, depending on the specific montage and physiological measure employed (Bikson et al., 2010). It should be noted that different activity of the reference electrode site produces different brain activity modulation of the target site. Therefore, most published studies used an alternative solution involving the placement of a large reference electrode at the contralateral orbita; this choice was made because the current under the large electrode is dispersed (Nitsche et al., 2007).

Mono-cephalic tDCS, is another common montage; it involves the placement of an extra-cephalic reference electrode for monocephalic stimulation. This approach helps eliminating the confounding effects of the reference electrode on the head. This protocol has been adapted by many studies with different montages and various placements of the extra-cephalic reference electrode. Placements of the electrode have included, for instance: M1 and ipsilateral shoulder (Accolla et al., 2007), inion and neck base (Accornero et al., 2007), left fronto-temporal areas or inion and right shoulder (Monti et al., 2008), bi-frontal and non-dominant arm (Koenigs et al., 2009), bilateral dorsolateral prefrontal cortex and right deltoid (Priori et al., 2008). However, safety concerns regarding the currents passing through the brainstem, which may produce nausea, respiratory difficulty, muteness, and impaired fine motor control have been raised (Lippold and Redfearn, 1964). Nevertheless, additional evaluations of the local electric field distributions generated by tDCS with an extra-cephalic reference electrode revealed that the brainstem's cardio-respiratory and autonomic centers were not modulated by an extracephalic reference electrode (Im et al., 2012).

**Figure 1:**
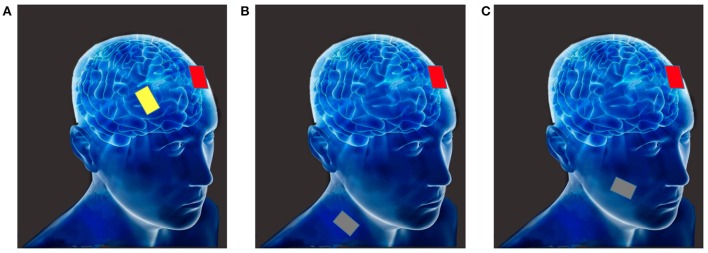
**(A)** Bi-cephalic (anode in red, cathode in yellow); **(B)** Mono-cephalic tDCS (active anode or cathode in red, the extra-cephalic reference electrode in gray); **(C)** Non-cephalic tDCS (active anode or cathode in red, the non-cortical reference electrode in gray).

The third type of tDCS montage is non-cephalic tDCS. In this montage the direct current stimulation is delivered to non-cortical brain areas. For example, Ferrucci et al. (2008b) stimulated the cerebellum with tDCS; they found that this stimulation impaired the practice-dependent elevation of the reaction times in a working memory task. Similarly, Galea et al. (2009) investigated the inhibitory role of the cerebellum on motor-evoked potentials (MEPs) elicited by transcranial magnetic stimulation (TMS) over the motor cortex, and revealed that MEPs could be modified by tDCS in a polarity-specific manner. Figure 1 depicts the above-discussed montages.

### Underlying mechanisms of tDCS

Although the exact underlying mechanisms of tDCS effects on cortical excitability have not been fully understood, two potential mechanisms were proposed (for reviews, see Arul-Anandam and Loo, 2009; Brunoni et al., 2012a). First, Arul-Anandam and Loo (2009) argued that anodal stimulation propelled neuronal resting membrane potential toward depolarization, while cathodal stimulation propelled resting membrane potential toward hyperpolarization. While this theory can explain the short-term effects of tDCS, it fails to provide an adequate explanation for the long lasting effect of tDCS. This limitation stems from the fact that one single session of tDCS could elicit long lasting after-effects (Nitsche and Paulus, 2001; Nitsche et al., 2003b) while changes in resting membrane potential are short-lived.

Second, it was suggested that tDCS could also induce synaptic changes by adjusting the strength of synaptic transmission, a mechanism similar to long-term potentiation (LTP) and long-term depression (LTD) (Nitsche et al., 2003c). For example, recent evidence from pharmacologic studies reported that the after-effects of anodal tDCS were considerably shortened by the injection of propranolol (PROP), a β-receptor antagonist. In other words, N-Methyl-D-aspartate (NMDA) receptor-dependent LTP could be modified by anodal tDCS (Stagg and Nitsche, 2011). Furthermore, Fritsch et al. (2010) demonstrated that brain-derived neurotrophic factor (BDNF; a neurotrophin that is important in late-phase LTP) was essential for the after-effects of direct cortical stimulation. The pharmacologic modulation of glutamatergic activity can contribute to the cathodal tDCS after-effects as well (Stagg and Nitsche, 2011). Ultimately, while the exact neural mechanisms underlying tDCS are largely unknown, the abovementioned theories provide possible accounts for what drives tDCS effects and its efficacy in achieving short- and long-term brain modulations.

### Advantages of tDCS over other invasive and non-invasive brain stimulation techniques

Compared to other cognitive neuroscience methods such as invasive brain stimulations (e.g., deep brain stimulation, epidural cortical stimulation), tDCS is presumed to be safer and cheaper. Invasive brain stimulation methods are associated with surgical risks (infection, acute inflammation, and/or seizures; Zaghi et al., 2009), and tend to involve more expensive medical labor (e.g., neurosurgeons). Like other manipulation techniques, but with minimal discomfort, low cost, and no invasion, tDCS can directly manipulate the brain function in regions of interest and thereby help examining the effect of those manipulations on behaviors or produce desirable transient activity changes. The research potential of tDCS is therefore in that it could find causal relationships between specific brain regions and behavioral changes. Furthermore, it can help pointing to needed brain regions for different cognitive, subconscious and behavioral brain processes, like lesion studies do, but without the permanent brain damage.

tDCS can also be advantageous compared to transcranial magnetic stimulation (TMS; another noninvasive technique used to influence neuronal activity). First, tDCS is relatively low cost, user-friendly, portable and tolerable (Priori et al., 2009; Zaghi et al., 2009; Brunoni et al., 2011a,b; Kessler et al., 2012). Second, tDCS can be easily combined with pharmacotherapy. For example, Brunoni et al. (2011c) have demonstrated that the combined use of tDCS and Sertraline (an antidepressant of the selective serotonin reuptake inhibitor class) requires decreasing dosage injects in clinical trials. Third, tDCS is relatively safe (safety will be discussed later in this manuscript), while TMS has been associated with the potential for causing seizures if inappropriately applied (Classen et al., 1995). Fourth, tDCS tends to have better experimental control because sham (placebo) stimulation is indistinguishable from real stimulation. Sham stimulation is administrated by increasing current over several seconds (typically 30 s) to the target brain region, and then fading off over several seconds. Using this procedure, subjects theoretically obtain the same experience as they do during real stimulation such as itching and tingling. The sensations are transient in real stimulation as well as in sham stimulation because subjects get used to the current in real stimulation, whereas the current tapers off in sham stimulation (Kessler et al., 2012). Finally, it may be easier to produce longer-lasting modulatory effects of cortical function with tDCS than with TMS (Fregni et al., 2006a). Overall, the advantages of tDCS over other invasive and non-invasive brain stimulation techniques make it an important technique that merits further research.

## tDCS applications in clinical samples

Given the above-mentioned advantages and capacities of tDCS, it is not only used as a tool for neuroscience research, but can also be applied for the assessment and treatment of various psychiatric and neurological disorders, including drug addiction, major depression disorder, and Alzheimer's disease. Table 1 describes the select studies discussed in this article.

**Table 1:** **Clinical studies of tDCS in the assessment and treatment of psychiatric and neurological disorders**.

**References**	**Participants**	**Age (years)**	**Anodal electrode**			**Cathodal electrode**		**Duration**	**Results**
			**Position**	**Area (cm^2^)**	**Intensity**	**Polarity Position**	**Area (cm^2^)**		
**MAJOR DEPRESSION DISORDER (MDD)**									
Conti and Nakamura-Palacios, 2014	13 cocaine addicts	23–37	F4^*^	35	2 mA	F3^*^	35	20 min	Prefrontal tDCS modulated the ACC response in crack-cocaine users.
Wang et al., 2016	20 male heroin addicts	29–57	Bilateral FPT	35	1.5 mA	Occipital lobe	35	20 min	tDCS over bilateral FPT area significantly reduced heroin addicts' craving score.
Falcone et al., 2016	25 smokers	18–60	F3^*^	25	1 mA	Right supra-orbital area	25	20 min	Active tDCS significantly increased latency to smoke and decreased the total number of cigarettes smoked during the session.
Shahbabaie et al., 2014	30 abstinent meth users	20–45	F4^*^	35	2 mA	Left supra-orbital area	35	20 min	Active prefrontal tDCS acutely reduced craving at rest in the abstinent methamphetamine users.
den Uyl et al., 2015	41 heavy drinkers	21.7 ± 2.8	right IFG/F3^*^	35	1 mA	Right supra-orbital area	35	10 min	DLPFC stimulation can reduce craving in heavy drinkers.
da Silva et al., 2013	13 alcoholic subjects	29–59	F3^*^	35	2 mA	Right supra-deltoid area	35	20-min, 5 sessions	Active tDCS significantly modulated craving induced by alcohol related cues and improved mood scores.
Gorini et al., 2014	18 dependent cocaine users	29–53	F3^*^/ F4^*^	32	1.5 mA	F4^*^/F3^*^	32	20 min per task	DLPFC (left and right) tDCS reduces risky behaviors at the BART task; Right DLPFC anodal tDCS increased safe behavior while left DLPFC anodal tDCS increased risk-taking behavior at the GDT task.
Meng et al., 2014	30 smokers	23.7 ± 7.2	FPT	33	1	Occipital lobe/FPT	33	20 min	Low current bilateral cathodal stimulation of the FPT area attenuates smoking cue-related attention and smoking behavior.
Bennabi et al., 2015	23 treatment resistant MDD patients	61.8 ± 16.3	F3^*^	35	2 mA	FP2^*^	35	30 min, 10 sessions	No difference in mood improvement, responder rate, or changes in neuropsychological tests.
Blumberger et al., 2012	24 treatment resistant MDD patients	18–65	F3^*^	35	2 mA	F4^*^	35	20 min, 15 sessions	No significant difference between active and sham tDCS.
Brunoni et al., 2011a	17 MDD patients & 14 BDD patients	30–70	Left DLPFC	35	2 mA	Right DLPFC	35	20 min, 10 sessions	Depressive symptoms in both MDD and BDD groups diminished, and the beneficial effect persisted at one week and one month.
Brunoni et al., 2013	103 MDD patients	18–65	F3^*^	25	2 mA	F4^*^	25	30 min, 10 sessions	Greater mood improvement after active tDCS + sertraline compared to all other groups. Active tDCS only was significantly superior to placebo, but no difference between active tDCS and sertraline taken solely.
Brunoni et al., 2014a	24 depressed patients	18–65	F3^*^	25	2 mA	F4^*^	25	20 min	A single bilateral DLPFC tDCS session could modify the negative attentional bias.
Brunoni et al., 2014b	37 MDD patients	46.1 ± 10.4	F3^*^	25	2 mA	F4^*^	25	30 min, 10 sessions	Greater mood improvement after active vs. sham tDCS only in older patients and those who presented better performance in the cognitive task.
		41.5 ± 10.6							
Rigonatti et al., 2008	42 MDD patients	49.4 ± 7.4	Left DLPFC	#	2 mA	Right supra-orbital area	#	20 min, 10 sessions	The antidepressant effects of tDCS are similar to those of a 6-week course of fluoxetine at a relatively small dose of 20 mg/day; however, the effect of tDCS appears to become significant faster than that of fluoxetine.
Wolkenstein and Plewnia, 2013	14 MDD patients	31.8 ± 9.8	F3^*^	35	1 mA	Right deltoid muscle	35	20 min	A single session of anodal tDCS over left DLPFC improves deficient cognitive control in MDD.
**ALZHEIMER'S DISEASE (AD)**									
Boggio et al., 2009	15 AD patients	77.5 ± 6.9	T3^*^ and T4^*^	35	2 mA	Right deltoid muscle	64	30 min, 5 sessions	Repetitive tDCS over temporal cortex significantly improved visual memory in AD patients.
		80.6 ± 9.5							
Boggio et al., 2012	10 AD patients	79.1 ± 8.8	F3^*^/T7^*^	35	2 mA	Right supra-orbital area	35	30 min, 3 sessions	Low current tDCS of DLPFC and LTC significantly enhanced AD patients' visual recognition memory performance.
Cotelli et al., 2014	36 AD patients	76.6 ± 4.6	Left DLPFC	25	2 mA	Right supra-orbital area	60	25 min, 10 sessions	No difference in cognitive performance improvement (face-name association task) after active vs. sham tDCS combined with memory training.
		74.7 ± 6.1							
		78.2 ± 5.2							
Ferrucci et al., 2008a	10 patients with probable AD	64–84	TPA	25	1.5 mA	Right deltoid muscle	25	15 min	tDCS delivered over the TPA can specifically affect recognition memory performance in patients with AD.
Khedr et al., 2014	34 AD patients	69.7 ± 4.8	Left DLPFC	24	2 mA	Right supra-orbital area	100	25 min, 10 sessions	Improvement in cognitive performance after either anodal or cathodal vs. sham tDCS intervention, with reduction of P300 latency.
Penolazzi et al., 2015	1 AD patients	60	F3^*^	35	2 mA	Right supra-orbital area	100	20 min, 10 sessions	The synergetic use of tDCS and CTs appeared to slow down the cognitive decline of AD patients.
Suemoto et al., 2014	40 AD patients	80.5 ± 7.5	Left DLPFC	35	2 mA	Right supra-orbital area	35	25 min, 6 sessions	No change in apathy scores, global cognition, and neuropsychiatric symptoms after active vs. sham tDCS.
**PSYCHIATRIC DISORDERS (ADHD, SCHIZOPHRENIA, DOC)**.									
Bandeira et al., 2016	9 children with ADHD	11.1 ± 2.8	F3^*^	35	2 mA	Right supra-orbital area	35	30 min, 5 sessions	Improvement in selective attention and inhibitory control after 5 daily repeated active tDCS intervention.
Cosmo et al., 2015	60 ADHD children	31.8 ± 11.6	F3^*^	35	1 mA	F4^*^	35	20 min	No significant differences between active vs. sham tDCS in the go/no-go task after a single 1mA 20 min tDCS intervention.
		32.7 ± 10.4							
Soltaninejad et al., 2015	23 children with ADHD symptoms	15–17	F3^*^	35	1.5 mA	FP2^*^	35	15 min	tDCS over the left DLPFC of adolescents with ADHD symptoms can improve inhibitory control.
Sotnikova et al., 2017	16 ADHD children	14.3 ± 1.3	F3^*^	13	1 mA	Cz^*^	35	20 min	After active vs sham TDCS, there was a signifcant effect on working memory; tDCS caused greater activation of the left DLPFC, the left premotor cortex, left SMA, and precuneus. Functional connectivity of working memory and executive control netwoks significantly increased.
Brunelin et al., 2012	30 schizophrenia patients	40.4 ± 9.9	Left DLPFC	35	2 mA	Left TPJ	35	20 min, 10 sessions	AVH were robustly reduced after active vs sham tDCS, which lasted for 3 months; The benificial effect on negtive symptoms was also observed.
		35.1 ± 7.0							
Mondino et al., 2015	28 schizophrenia patients	36.5 ± 9.6	Left DLPFC	35	2 mA	Left TPJ	35	20 min, 10 sessions	Compared to sham tDCS, active tDCS significantly reduced covert/overt speech misattributions and AVH frequency.
		39.2 ± 9.0							
Mondino et al., 2016	23 schizophrenia patients	36.7 ± 9.7	Left DLPFC	35	2 mA	Left TPJ	35	20 min, 10 sessions	Relative to sham tDCS, active tDCS signifcantly reduced AVH as well as the negative symptoms. The reduction of AVH severity was correlated with the reduction of the rs-FC between the left TPJ and the left anterior insula.
		37.3 ± 9.7							
Angelakis et al., 2014	10 DOC patients	19–62	F3^*^/C3^*^	25	1mA/2 mA	FP2^*^	35	1 mA: 20 min, 5 sessions; 2 mA: 20 min, 5 sessions	MCS but VS patients showed clinical improvement immediately after treatment.
Bai et al., 2017	16 DOC patients	17–68	F3^*^	25	2 mA	FP2^*^	25	20 min	tDCS can effectively modulate the cortical excitability of DOC patients, and the changes in excitability in temporal and spatial domains are different between patients with MCS and those with VS.
Estraneo et al., 2017	7 VS patients & 6 MCS patients	18–83	F3^*^	35	2 mA	FP2^*^	35	20 min, 5 sessions	No significant short-term clinical and EEG effects were found in patients with prolonged DOC after 5 daily repeated tDCS intervention.
Thibaut et al., 2014	25 VS patients &30 MCS patients	42 ± 17	F3^*^	35	2 mA	FP2^*^	35	20 min	tDCS over left DLPF cortex may transiently improve signs of consciousness in MCS but VS patients following severe brain damage.
		43 ± 19							
**CHILDREN WITH PSYCHIC DEVELOPMENT DISORDERS**									
Auvichayapat et al., 2013	22 focal epileptic children	6–15	Epileptogenic focus	35	2 mA	Contralateral shoulder	35	20 min	Decreased frequency of seizures; tDCS was safe, at least in the short term, for children with epilepsy.
Kessler et al., 2013	2 healthy children	8–12	C3^*^/F3^*^/…	25	0.5/1/2 mA	C4^*^/F4^*^/…	25	#	Results do not show that applying 2 mA of current is unsafe in children.
Mattai et al., 2011	12 COS children	10–17	bilateral DLPFC	25	2 mA	bilateral STG	25	20 min, 10 sessions	A 20-min duration tDCS of 2 mA of bilateral anodal and bilateral cathodal DC polarization to the DLPFC and STG was well tolerated in the COS population with no serious adverse events.
Pinchuk et al., 2012	128 LDC and 48 mild MRC	8–12	FP2^*^/mlFC	2.5/6.25	0.06–0.12 mA	Ipsilateral mastoid	6.25	18-45 min, 5–9 sessions	Practically no adverse effects; Improved mental functions in patients.

* *According to 10–20 EEG system. #, not mentioned; DLPFC, dorsolateral prefrontal cortex; M1, primary motor cortex; FPT, frontal-parietal-temporal association area; TPA, temporoparietal area; ADHD, attention defcit/hyperactivity disorder; DOC, disorder of consciousness; VS, vegetative state; MCS, minimally conscious state; AVH, auditory verbal hallucinations; COS, children with childhood-onset schizophrenia; LDC, Learning disorder children; MRC, mild mental retardation*.

### tDCS use in drug addiction treatment

Addiction is a persistent psychological state in which there is diminished capacity to control compulsive substance (such as drugs) or non-substance (such as gambling, social media) seeking, regardless of the risks and negative consequences associated with this behavior (Hyman and Malenka, 2001). To date, available treatments for addictive behaviors remain limited, and especially the long-term success rates have been fairly poor (O'Brien, 2011). For example, behavioral interventions, such as cognitive-behavioral therapy, showed modest benefits (Dutra et al., 2008), perhaps because many addicts have prefrontal cortex damage that renders cognitive approaches as less efficacious (Turel et al., 2014; He et al., 2017).

Given its potential capabilities, tDCS has attracted the interest of therapists as a potential tool for reducing drug craving and related addiction problem. Researchers have shown that repeated tDCS could modulate cortical excitability and thereby improve the neuronal activity of circuits associated with cognitive control over drug craving, thus alleviating drug addiction problems. Specifically, the dorsolateral prefrontal cortex (DLPFC) is involved in inhibitory control and planning; it is therefore a critical brain region for controlling compulsive drug-seeking and avoiding relapse (Janes et al., 2012; Yuan et al., 2016). Studies have suggested that enhanced activity in the DLPFC is associated with reduced craving (McBride et al., 2006).

Several researchers have therefore examined the possibility that the modulation effect of tDCS applied to the DLPFC helps alleviating drug addiction problems. For example, Falcone et al. (2016) investigated the effect of a single 20-min session of 1.0 mA anodal stimulation over the left DLPFC on 25 smokers. They found that the stimulation could improve their ability to resist smoking. Similarly, repeated anodal tDCS over the left DLPFC could also effectively reduce craving and prevent relapse in alcoholics and methamphetamine abusers (da Silva et al., 2013; Shahbabaie et al., 2014; den Uyl et al., 2015), and decrease risky choices in cocaine addicts (Gorini et al., 2014). Nakamura-Palacios et al. (2016). It is suggested that this cognitive improvement might be associated with ventromedial prefrontal cortex (VMPFC) activation as modulated by the DLPFC activity change that resulted from repetitive anodal tDCS. The indirect modulation of VMPFC activity is therefore argued to be the means through which tDCS produces better self-control in addicts, improved decision making and reduced risk of relapse.

The bilateral frontal-parietal-temporal association area (FPT) is another potential target of tDCS for treating addictions. For example, Meng et al. (2014) examined the effects of inhibiting the FPT on the attentional bias to smoking-related cues in smokers. They found that low current bilateral cathodal stimulation of the FPT attenuates smoking cue-related attention; it hence can reduce desire to smoke in response to smoking cues in the environment. Similarly, Wang et al. (2016) found that one single tDCS session (1.5 mA for 20 min) over the bilateral FPT reduces cue induced craving in heroin addicts.

These studies suggest that transient and repeated anodal tDCS of DLPFC or cathodal over FTP could significantly improve the ability to resist drug craving or directly reduce patients' subjective cravings. However, knowledge regarding the neural mechanisms underlying such effects is still limited. Future studies should either combine pharmacologic antagonists with tDCS or image the brain before and after tDCS stimulation to better understand the neurochemical and neuro-network changes induced by tDCS and its resultant therapeutic effects.

### tDCS use in major depressive disorder treatment

Major depressive disorder (MDD) is a complex and inhomogeneous mental disorder, which is characterized by a pervasive and persistent low mood and usually accompanied by alterations of cortical excitability. The neural system hypothesis suggests that depression is a disorder related to dysfunction in structures implicated in the experience of emotions and the processing of affective information and stimuli; these include the dorsolateral and ventral prefrontal cortex, hippocampus and amygdala (Hamilton et al., 2008).

Since tDCS can modulate cortical excitability for hours after the stimulation, it was proposed that it might be a promising option for treating depression patients, beyond the use of psychotherapeutic interventions and pharmacotherapy. Numerous neuropsychological studies have revealed that stimulation of the DLPFC with tDCS can act in depression patients via re-establishing the balance between left and right prefrontal cortices, i.e., by enhancing the left hypoactive DLPFC activity and reducing the excitability of the right hyperactive DLPFC. For instance, Brunoni et al. (2014a) found that one single active bi-frontal tDCS significantly modifies negative attentional bias in MDD, which is consistent with the conclusion of Wolkenstein and Plewnia (2013) that anodal tDCS over the left DLPFC improved deficient cognitive control in MDD.

Extending the single session effect, the long-term effect of tDCS on MDD patients have also been established by Brunoni et al. (2011a). They found that after 20 min, twice daily, 5-day tDCS, depressive symptoms in all patients with MDD and bipolar depressive disorder (BDD) diminished, and that the beneficial effect persisted for 1 week and 1 month, respectively. Furthermore, Rigonatti et al. (2008) compared the clinical effects of PFC tDCS and the antidepressant fluoxetine in MDD patients; they found that active tDCS and fluoxetine both reduced depressive symptoms considerably.

Several less promising tDCS studies involving MDD patients were also reported (Blumberger et al., 2012; Bennabi et al., 2015). For examole, Blumberger et al. (2012) sought to characterize the pattern of change in depressive symptomatology induces by anodal tDCS to the left prefrontal cortex. Results showed that tDCS did not induce clinically relevant antidepressant effect in 24 treatment-resistant depressed patients. However, the low efficacy demonstrated in these tDCS studies may be attributed to the patients' strong psychological resistance to treatment, especially considering the fact that the same tDCS protocol showed significant antidepressant efficacy in MDD patients with low treatment resistance (Brunoni et al., 2011a, 2013, 2014b). It was also demonstrated that moving the right frontal cathodal electrode to an extra-cephalic position (e.g., right upper arm) may result in a better therapeutic effect in treatment-resistant patients (Martin et al., 2011). Thus, non-significant effects may be due to boundary conditions of tDCS and montage choices.

Taken together, these results have clearly showed a potential anti-depressive effect of prefrontal tDCS via altering the function of emotion-related information processing circuits. Specifically, the current approach is to enhance neural activity of left DLPFC and/or reduce neural activity of right DLPFC via the two main protocols of anodal tDCS over left DLPFC with the cathode over right DLPFC or right OFC (Brunoni et al., 2012b). It should be noted, however, that tDCS efficacy on specific symptom profiles in pharmacotherapy-resistant depression is limited (Bennabi et al., 2015), especially via the protocol of the left DLPFC anode with right OFC cathode (Lefaucheur et al., 2017). Nevertheless, such studies have noteworthy limitations, including relatively small sample sizes, and future studies should focus on the treatment effects of tDCS in larger and more representative samples of MDD patients. There is still a long way to go in the optimization of tDCS parameters in order to get better and more consistent therapeutic effects; current studies in this area fail to show that tDCS is a significantly better treatment than pharmacotherapy in MDD cases. Examining these competing (and possibly supplementary) approaches is a promising avenue for future research.

### tDCS use in Alzheimer's disease treatment

Alzheimer's disease (AD) is a neurodegenerative and progressive disease; it manifests through severe general cognitive decline in memory, attention, language, and executive control functions. Although in 2015 there were over 46.8 million people worldwide who suffer from AD, much is still to be learned about the neural underpinnings, pathogenesis and etiology of AD (Prince, 2015). The AD-Amyloid hypothesis provides an account for one possible biological mechanism underlying AD development. It proposes that the development of AD is driven by the accumulation and deposition of extracellular amyloid beta peptide aggregates in the brain and that such depositions can trigger a cascade that harms neurons and synapses (Kung, 2012). Another account from a neuropathology perspective, suggests that AD is caused by loss of neurons and synapses in the cerebral cortex and key regions for cognitive functioning, such as the temporal lobe, parietal lobe, frontal lobe and cingulate gyrus (Wenk, 2003). Attempts to treat AD with pharmacology have so far produced limited effectiveness or controversial outcomes (Nardone et al., 2011; Piau et al., 2011).

Non-pharmacological interventions, such as tDCS, have been recently found to produce positive effects for the treatment of older adults with memory decline or dementia (Elder and Taylor, 2014; Hsu et al., 2015). Moreover, researches have demonstrated that the aging brain can still morph, reorganize and change (Kuo et al., 2013; Gutchess, 2014). Hence, it is possible that neuro-stimulation like this produced by tDCS could influence ameliorate-specific parts of the brain, as well as the anti-correlated functional networks of the brain in order to control ameliorate effects (Li et al., 2003; Hui et al., 2009). Studies have shown significant changes in brain activity in the medial temporal lobes and temporoparietal cortex (TPC) during a memory task in AD patients (Rémy et al., 2005). Consequently, several studies explored the practical effects of tDCS on such regions in AD patients (Ferrucci et al., 2008a; Boggio et al., 2009, 2012). For example, Ferrucci et al. (2008a) evaluated the effect of tDCS over the temporopolar cortex (TPC) in 10 patients with AD. They found that all patients after an active anodal tDCS over the TPC area showed a significant improvement in recognition memory performance rather than visual attention, suggesting that the effects of tDCS over TPC were likely specific for recognition memory. Similarly, Boggio et al. (2009) assessed 10 AD patients with a memory recognition task using anodal tDCS over the left temporal cortex and left DLPFC, respectively. They found a significant positive effect of tDCS stimulation on short-term memory (visual recognition memory). In addition, They further examined the long-term effect of anodal tDCS over the temporal cortex on a visual recognition memory task in 15 AD patients, after five consecutive sessions. They found that AD patients' performance on the visual recognition memory task improves and that the improvement persists for at least 4 weeks after therapy (Boggio et al., 2012).

Such cases have substantiated the positive effect of tDCS stimulation on AD patients. They therefore suggest that tDCS may be an alternative or supplementary therapeutic option for AD patients. Later studies attempted to combine tDCS with cognitive training (CT), trying to produce constant and long-lasting curative effect. For instance, Penolazzi et al. (2015) tested the cognitive effects of two cycles of tDCS with CT treatments (Active tDCS + CT), 2 months apart. They found that the “tDCS + CT” could stabilize the global cognitive functioning for approximately 3 months, longer than the effect produced by CT only. Therefore, the synergetic use of tDCS and CT appeared to slow down the cognitive decline in AD patient, which again points to the potential value of tDCS as an adjuvant tool for cognitive rehabilitation in AD patients.

Although the efficacy of tDCS over the temproal cortex in improving recognition memory has been confirmed by several studies, researches have tried to explore the possibility thet the left DLPFC can be a potential stimulation target for ameliortaing AD patients' cognitive functions. For example, Khedr et al. (2014) investigated the long-term efficacy of anodal tDCS over the left DLPFC (2 mA, 25 min, and 10 days) in the neurorehabilitation of AD. The results showed that AD patients' cognitive performance significantly improved after either anodal or cathodal vs. sham tDCS, with reduction of P300 latency as the objective biological marker of AD. However, the same protocol (anodal tDCS over the left DLPFC, 2 mA, 25 min) applied by Cotelli et al. (2014) and Suemoto et al. (2014) showed negative results.

In summary, recent studies suggest a possible positive effect of tDCS on cognitive functions in AD patients, especially when the tDCS stimulation is combined with CT. Based on current evidence, it seems that the left TPC/temporal cortex may be a better target for tDCS stimulation compared to the left DLPFC, especially when researchers aim at improving AD patients' cognition memory. Future research should pay closer attention to the potential integration of tDCS with traditional methods such as pharmacological treatment and psychotherapy for improving cognitive rehabilitation in AD patients. Moreover, ways for extending the tDCS effects should be explored, because currently 1 month is the limit, and this is too short for meaningful rehabilitation of AD patients. Future studies should also try to understand the mechanisms underlying long-term effects of tDCS, as tDCS might interact with mechanisms involved in neurodegeneration with either beneficial (delayed deterioration of cognition) or harmful effects (accelerated cognitive deterioration) (Hansen, 2012).

### tDCS use in treating other psychiatric disorders

tDCS has also been applied to treat many other psychiatric and neurological disorders, beyond substance addiction, Alzheimer's disease and MDD. In the following paragraphs, we focus on select recent tDCS-related trends and new directions in the fields of ADHD (Sotnikova et al., 2017), schizophrenia (Pondé et al., 2017), disorders of consciousness (Cavaliere et al., 2016) and others.

#### ADHD

Attention deficit hyperactivity disorder (ADHD) is a persistent pattern of inattention and/or hyperactivity-impulsivity that interferes with functioning or development, has symptoms present in two or more settings (e.g., at home, school, or work; with friends or relatives; in other activities), and directly negatively influences social, academic or occupational functioning (American Psychiatric Association, 2013). The exact pathophysiology of ADHD has been difficult to delineate because of complicating factors such as evolving diagnostic criteria, phenotypic heterogeneity, frequent comorbidities, and environmental variables that may exacerbate or mimic symptoms (Rubio et al., 2016). One of the most influential theories for the neural basis of ADHD has focused on deficient inhibitory control leading to executive dysfunction (Gilbert et al., 2011), which has been proved to be linked to frontostriatal circuits, specifically hypo-activity in the DLPFC, ventrolateral prefrontal cortex, and the anterior cingulate cortex (Konrad et al., 2006). Thus, some studies have investigated the theraputic effect of tDCS over the DLPFC (Cosmo et al., 2015; Soltaninejad et al., 2015; Bandeira et al., 2016; Sotnikova et al., 2017). Although, several studies showed that tDCS over the left DLPFC of ADHD patients can improve executive control functions (e.g., inhibitory control, processing speed, working memory) and alleviate ADHD symptoms (Soltaninejad et al., 2015; Bandeira et al., 2016; Sotnikova et al., 2017), Cosmo et al. (2015) did not find significant differences between active and sham bihemispheric tDCS of the DLFPC in terms of improved inhibitory control.

#### Schizophrenia

Schizophrenia is a chronic mental disturbance characterized by dysfunction in emotion, cognition, and perception of reality (Kuo et al., 2014). Clinical features of schizophrenia include psychotic/positive symptoms (e.g., hallucinations, delusions, and disturbances of thought) and negative symptoms (e.g., emotional dullness, anhedonia, alogia, or attention deficit). The primary objective for tDCS in schizophrenia patients has been to reduce auditory verbal hallucinations (AVH). Thus, left temporo-parietal junction (TPJ) may be a promising cathodal target (Brunelin et al., 2012). On the other hand, numerous studies have revealed that the impaired frontal cortical activity may underlie the cognitive dysfunction in schizophrenia. Given the pivotal role of the DLPFC in mediating a wide range of executive functions (Niendam et al., 2012), this region can be the anodal region of interest. For example, Brunelin et al. (2012) showed a significant reduction of AVHs and positive and negative symptoms following active tDCS (anode: the left DLPFC; cathode: left TPJ) compared to sham condition. This is similar to the conclusion of Mondino et al. (2015, 2016), who found that the functional connectivity of left TPJ with the left angular gyrus, the left DLPFC and the precuneus increased following active tDCS. These regions are involved in the language-related and self-other recognition network (Mondino et al., 2014).

#### Disorders of consciousness

The neural basis, clinical recognition, and long-term outcome of disorders of consciousness (DOC), such as coma, minimally conscious state (MCS) and vegetative state (VS), remain poorly understood (Giacino et al., 2014). Although peripheral treatments (e.g., physical therapy, speech therapy) and many potential pharmacological interventions have been evaluated in recent years, there is still no unanimously accepted evidence-based treatment guidelines for DOC patients (Gosseries et al., 2011). Some studies explored the potential of tDCS to improve the patients' consciousness. Thibaut et al. (2014) found that tDCS over left DLPF cortex may transiently improve signs of consciousness in MCS, but not VS patients following severe brain damage, which is similar to Angelakis et al. (2014). Moreover, electrophysiological evidence revealed that tDCS can effectively modulate the cortical excitability of patients with DOC. They further revealed that the changes in excitability in temporal anspatial domains are different between patients with MCS and those with VS (Bai et al., 2017). However, Estraneo et al. (2017) found that repeated tDCS did not exert remarkable short-term clinical and EEG effects in patients with prolonged DOC. In conclusion, some beneficial results of tDCS protocols have been demonstrated in DOC patients, especially targeting the left DLPFC in MCS. However, the reported studies are preliminary, were based on small samples, and used heterogeneous outcome measures, including either clinical or functional connectivity variables (Lefaucheur et al., 2017). Hence, further studies should examine the long-term therapeutic effects of tDCS over the left DLPFC in DOC patients.

## Possible adverse effects and safety procedures

### Possible detrimental effect of tDCS

Although tDCS has been recognized widely as a safe brain stimulation technique, there are still some potential detrimental effects worthy of mentioning and considering. First, researchers and therapists should pay attention to skin lesions that usually occur under the anodal or cathodal electrodes in patients after treatment with tDCS. Specifically, the skin temperature increase under the electrodes and chemical reactions in the interface of the skin and electrodes might cause skin lesion. These lesions may be associated with several factors. Specifically, the degree of skin lesions was found to be positively related to stimulation intensity, skin impedance (Palm et al., 2008), duration of stimulation, accumulation of electrochemically produced toxins, and the dissolution products of the sponges (Frank et al., 2010). This happens, in part, because skin impedance can change as a function of current intensity, density of stimulations and stimulation duration (Kalia and Guy, 1995; Prausnitz, 1996). It was also suggested that electrode impedance might change depending on the dynamic electrochemical processes as well as the stimulation waveform and time (Prausnitz, 1996; Merrill et al., 2005; Minhas et al., 2010). Therefore, to use tDCS safely, the stimulation voltage should be adjusted to maintain the desired current magnitude across variable impedances.

Second, in addition to skin lesions, it is also worth mentioning that tDCS can be associated with a few mild adverse effects, such as mild tingling, mild pain, and transient redness (Iyer et al., 2005; Fregni et al., 2006c; Poreisz et al., 2007; Plazier et al., 2012). Such adverse effects have been observed under various tDCS montages and protocols and over different cortical areas in healthy participants, as well as in patients with various neurological disorders (Poreisz et al., 2007). For example, a recent study investigated the prevalence of side-effects in a cohort of 102 subjects with a total of 567 tDCS sessions (1 mA). The results showed that side effects can include a mild tingling sensation (70.6%), moderate fatigue (35.3%), a light itching sensation (30.4%), headache (11.8%), nausea (2.9%), and insomnia (0.98%) (Poreisz et al., 2007). However, these side effects seem to be safe, transient and could be tolerated well by most subjects (Nitsche et al., 2008; Loo et al., 2009; Miranda et al., 2009; DaSilva et al., 2011).

### Recommended safety procedures

In order to ensure that participants undergo safe tDCS stimulation, several procedures are recommended. First, researchers and therapists should have proper exclusion criteria, such as the presence of metallic implants in the skull or brain and skin diseases (Utz et al., 2010). Second, subjects' skin should be lightly cleaned with a swab, and researchers and therapists should avoid abrading the skin because eroded skin is prone to induce skin lesions. The static impedance of the skin should also be measured, and stimulation should not be carried out unless impedance levels are within limits according to the tDCS device manufacturer.

Third, electrode sponges should be soaked with saline solution to minimize skin resistance and ensured that the contact with the skin is sufficiently firm. Fourth, constant current strength should be provided by the stimulation device. A safety current density limit of 0.029–0.142 mA/cm^2^ (Poreisz et al., 2007) as well as a maximum charge density of 40 μC/cm^2^ at the stimulating electrode (Yuen et al., 1981; Agnew and McCreery, 1987) are suggested for a safely delivered tDCS. Fifth, for repeated applications of tDCS, a sufficiently large interval between sessions is suggested in order to avoid undesirable cumulative effects. The interval is determined by the stimulation purpose and procedure. Most importantly, the experimenter or therapist should be well prepared and oversee the whole procedure, especially when examining children and patients. Lastly, all other safety measures suggested by the device manufacturers and/or the lab should be followed.

Compared to adults, children's or adolescents' brains are still developing, and hence are prone to relatively stronger neural plasticity. Therefore, tDCS in children may theoretically lead to some unexpected or even dangerous results, such as brain tissue lesions and cognitive impairment. As such, it has been recommended that brain stimulation for pure cognitive enhancement should be delayed until the patient has reached the state of intellectual maturity (Maslen et al., 2015). However, since tDCS can produce positive results in various psychiatric patients, the use of tDCS among children and adolescents who need treatment has been explored (Fiocchi et al., 2016; Gschwind and Seeck, 2016; Muszkat et al., 2016; Palm et al., 2016).

The most important issue to be considered when administrating tDCS in children is the dosage. While tDCS is widely used in adults, the dosage of tDCS administration in children is poorly understood. It is still unknown whether stimulation parameters used in adults are also suitable for children to achieve similar results (i.e., safety and efficacy concerns; Kessler et al., 2013). Several studies tried to explore the safety of tDCS in children with neurological or psychiatric disorders. For instance, Auvichayapat et al. (2013) examined the seizure reduction effects as well as the safety of tDCS in children with refractory epilepsy. Results showed that tDCS could decrease the frequency of seizure. Based on this, they argued that tDCS, if used for short periods was safe for children with epilepsy (1 session of 1 mA cathodal tDCS for 20 min, 35 cm^2^). Similarly, Mattai et al. (2011) explored the safety and tolerability of tDCS in children with childhood-onset schizophrenia and found that the children tolerated tDCS well with no serious side effects (10 sessions of 2 mA tDCS for 20 min, 25 cm^2^). Moreover, Pinchuk et al. (2012) found significant improvement of higher cognitive functions when stimulating children with psychic development disorders (5–9 session of 0.06–0.12 mA, 25–45 min, 2.5–6.25 cm^2^). Specifically, verbal functions improvement was observed in 80% of the children, and writing mistakes were reduced 3-fold in patients with dysgraphia.

It should be noted that the above results primarily focus on childhood neurological or psychiatric disorders. To date, the safety procedures for, and adverse effects of tDCS in healthy children still requires further research.

## tDCS combined with other techniques

### tDCS and MRI

Combining magnetic resonance imaging (MRI) with concurrent tDCS allows for a non-invasive examination of tDCS-induced effects throughout the brain. However, the potential risks and technical issues caused by wires and electrodes within the MRI scanner should be noted. They are similar to those discussed regarding electroencephalography (EEG) concurrent recording within the MRI. Undesired coupling of the tDCS wires and the MRI transmission coil could burn the skin as well as distort flip angles. Nevertheless, with specially manufactured MR-compatible electrodes, there is a growing body of evidence indicating that specific tDCS tools can be safely and effectively used in the MR environment. For instance, Kwon et al. (2008) demonstrated the modulation effect of tDCS on the primary motor cortex by functional MRI. Similarly, Antal et al. (2011) explored the online effects of short periods of tDCS on the brain activity as well as the associated hemodynamics by concurrent BOLD fMRI. No distortion, signs of elevated flip angles, or signal loss of the brain images near the electrodes were evident in their study, and no brain signal changes were observed when turning the direct current stimulation on and off (Zheng et al., 2011). This suggests that the combination of tDCS and functional MRI could be applied as a safe method, even in a 3T MR environment; though caution regarding such a combination should be applied in future applications.

### tDCS and TMS

tDCS may be more valuable if combined with other neuro-modulatory approaches such as TMS. With respect to tDCS-TMS studies, Lang et al. (2004) reported that a priming effect occurred when tDCS was followed by repetitive TMS. In this study, 100 stimuli of 5 Hz were employed in a repetitive TMS procedure, after an anodal, cathodal or sham tDCS was applied to left M1 for 10 min. The study found that cathodal and anodal tDCS increases and decreases the subsequent effects on corticospinal excitability, independently. Similar priming effects occurred when tDCS was followed by repetitive TMS (Siebner et al., 2004; Cosentino et al., 2012; Moloney and Witney, 2013). Furthermore, no adverse effects on neuropsychological function was found after repeated sessions of combined tDCS-rTMS stimulation (Loo et al., 2009). Thus, it appears that tDCS-TMS studies are safe, but the lead-lag effects of one on the other require attention and caution.

### HD-TDCS

High-Definition tDCS (HD-tDCS) is a newly developed technique using configurations of smaller, specially designed electrodes to improve the spatial focality of tDCS (Minhas et al., 2010). Various configurations of HD-tDCS have been proposed; these can be modified to improve the spatial focality of the stimulated brain regions. HD-tDCS uses deployments of specialized compact scalp electrodes to pass currents without inducing skin irritation and with minimal discomfort (Lang et al., 2005). Several clinical studies have been conducted in healthy subjects, as well as in patients to investigate its safety and efficacy. For instance, Borckardt et al. (2012) evaluated whether HD-tDCS over the motor cortex would reduce pain and sensory experience with the 4 × 1 ring deployment. They found that real HD-tDCS can decrease the heat and cold sensory thresholds and the thermal wind-up pain. They also observed a marginal analgesic effect for cold pain thresholds. In addition, Villamar et al. (2013) examined the HD-tDCS effects on overall perceived pain in fibromyalgia patients using the 4 × 1 ring configuration. They found that, compared to sham stimulation, both the anodal and cathodal stimulation led to significant decrease in overall perceived pain. He et al. (2016) used similar HD-tDCS configurations to investigate the effects of left and right DLPFC on risky decision making, and found that stimulating the left but not the right DLPFC showed higher performance in the Iowa Gambling task and lower delay discounting rate in healthy male participants. The reported effects of HD-tDCS were mild tingling or itching during both real and sham stimulation, which typically faded away after a few minutes. Hence, HD-tDCS seems to be as safe as tDCS, but future research is needed to further establish its efficacy and safety.

## Future directions

To date, based on the available evidence, tDCS can be treated as a relatively safe and portable brain activity modulation technology. It has been recognized as an effective neuromodulation tool for the treatment of neuropsychiatric disorders and for improving cognitive functioning in healthy subjects. No hard evidence that tDCS can damage brain tissue or impair cognitive functions has been produced. However, many challenges for the clinical practice of tDCS still need to be overcome and require further studies. For example, the effect of prolonged or repeated exposure to tDCS should be investigated in longitudinal designs. The possibility that the excitability of cortical neurons could be modulated by tDCS on a long-term basis should be studied (Mattai et al., 2011). Researchers should also explore and verify the long-term morphological changes or behavioral alterations after tDCS. Brain development of children is associated with intensive plasticity as well as other processes such as synaptic pruning. Therefore, researchers should investigate the long-term behavioral, brain structural and functional changes associated with tDCS in children and adolescents, which may provide valuable information for evaluating the therapeutic effectiveness of the stimulation protocol and its possible side effects (Kadosh et al., 2012).

Another important issue for future research is the mechanisms of action of tDCS. These mechanisms may have considerable significance for future clinical studies. Specifically, the mechanisms possibly involve various synaptic and non-synaptic effects on neurons, and non-neuronal cells and tissues of the central nervous system (Brunoni et al., 2012a), and these may shed light on the efficacy of combining tDCS with other therapeutic approaches, such as pharmacology. A better understanding of the tDCS mechanisms of action is also important for improving the clinical safety and efficacy of tDCS.

This improved understanding can be achieved by studying the brain mechanisms through combined techniques such as TMS, EEG, and fMRI. In addition, to increase the understanding of the biological and physiological etiology of tDCS stimulation effects, more elaborated experimental studies may be warranted in future, including animal studies *in vivo*. These can be useful, because critical mechanistic insights could be obtained from animal studies examining different tDCS montages or investigating other outcomes which could not be done in human, such as studies exploring the expression of neurotransmitter receptors. Such studies would facilitate translational tDCS research and have potential clinical implications (Brunoni et al., 2012a). Furthermore, these future animal studies might possibly open the door for certain biomarkers such as endophenotypes (Hasan et al., 2013).

Researchers should also attempt understanding the optimal treatment “dose” in tDCS. The concept “dose” of tDCS was first introduced by Nitsche and Paulus (2000). Nowadays, tDCS is known for its flexibility to have various dose options. At the same time, various dose options also make the optimal choice hard to ascertain. Cortical regions exposed to higher doses of tDCS may be more likely to be modulated. However, other less controllable factors such as skin and skull resistance may also have an influence on the amount of current that effectively reaches neuronal tissue. Current amount and delivery would be also modulated for patients with skull defects and brain lesions among other conditions. Hence, subjects with different conditions may get non-uniform effect by the same amount of current (Brunoni et al., 2012a). Future studies should investigate these factors and find the optimal doses and delivery approaches for safe and efficacious treatment among different populations of subjects.

Moreover, it should be noted that tDCS is not a catholicon of psychiatric diseases, even though it offers promising and exciting possibilities for treatment development. Given that multiple factors may influence the final clinical efficacy of tDCS in psychological and neurological disorders, further studies should focus on individual differences (e.g., psychological states, treatment resistance, and illness severity) in the specific disorder. As mentioned above, the degree of treatment resistance may hinder the therapeutic effect of tDCS in MDD (Mondino et al., 2014), and the illness severity can be an indication of therapeutic effect of tDCS in DOC patients (Angelakis et al., 2014; Thibaut et al., 2014); Psychological states of participants at the time of stimulation may also disturb the clinical outcomes. For example, tDCS will intensify but weaken addicts' cravings when the drug-related cues are present (Shahbabaie et al., 2014).

In addition, further studies should focus on study design and its crucial role in establishing and determining treatment outcomes of tDCS. The majority of studies with negative results were randomized controlled trials, which raises the possibility of a placebo effect. That is, sham tDCS may exert some degree of influence over outcomes (Kekic et al., 2016).

Lastly, it is interesting to consider the possibility of expanding the use of tDCS as an investigative tool for studying decision making in normal subjects, as well as in subjects with sub-clinical conditions. Since tDCS can manipulate the activity of brain regions involved in risk assessment, reflection and decision making (e.g., the insular cortex and/or DLPFC), it can be used for obtaining new insights in various branches of decision neuroscience, such as in neuro-marketing, neuro-information systems (Neuro-IS) and neuro-finance. In such research areas, the use of tDCS can help researchers pinpoint the neural regions that are involved in decision making tasks and how changes to the activation of these regions improves or impairs various decision making facets.

## Author contributions

HZ, LQ, and QH were responsible for the study conception and design; HZ, LQ, and DF wrote the first draft of the paper. SZ, OT, and QH also contributed to the writing of the paper. QH, SZ, OT, GX, JL, YL, and AC made the critical revision of the article. All authors gave the final approval of the article.

### Conflict of interest statement

The authors declare that the research was conducted in the absence of any commercial or financial relationships that could be construed as a potential conflict of interest. The reviewer CC declared their share affiliation and past co-authorship with the authors JL and GX, to the handling Editor, who ensured that the process met the standards of a fair and objective review. The reviewer JL declared their share affiliation with one of the authors YL to the handling Editor, who ensured that the process met the standards of a fair and objective review.
